# Synephrine Inhibits Oxidative Stress and H_2_O_2_-Induced Premature Senescence

**DOI:** 10.1007/s12013-025-01669-7

**Published:** 2025-01-20

**Authors:** Hiroshi Abe, Hiroko P. Indo, Hiromu Ito, Hideyuki J. Majima, Tatsuro Tanaka

**Affiliations:** 1 Department of Maxillofacial Radiology, Field of Oncology, Kagoshima University Graduate School of Medical and Dental Sciences, Kagoshima, 890-8544 Japan; 2 Quantum RedOx Chemistry Team, Quantum Life Spin Group, Institute for Quantum Life Science (iQLS), National Institutes for Quantum Science and Technology (QST), Chiba, 263-8555 Japan; 3 School of Allied Health Sciences, Walailak University, Nakhon Si Thammarat, 80160 Thailand

**Keywords:** Synephrine, Stress-induced premature senescence (SIPS), ROS, Mitochondrial dysfunction

## Abstract

Synephrine, a protoalkaloid found in *Citrus aurantium* (CA) peels, exerts lipolytic, anti-inflammatory, and vasoconstrictive effects; however, its antioxidant activity remains unclear. In this study, electron spin resonance spectroscopy revealed that synephrine scavenged both hydroxyl and superoxide anion radicals. Several external stimuli, such as H_2_O_2_, X-rays, and ultraviolet (UV) radiation, cause stress-induced premature senescence (SIPS). As oxidative stress induces SIPS, we hypothesized that synephrine, an antioxidant, would suppress H_2_O_2_-induced premature senescence in WI-38 cells. Synephrine significantly decreased the reactive oxygen species levels induced by H_2_O_2_, thereby reducing lipid peroxidation, and oxidative DNA damage and preventing SIPS. Additionally, synephrine inhibited mitochondrial dysfunction in H_2_O_2_-treated WI-38 cells. The expression levels of p53, p21, and p16^-INK4A^, which are involved in the induction of cell cycle arrest in SIPS, were significantly lower in synephrine-treated cells than in untreated cells. Our results indicate that synephrine inhibits H_2_O_2_-induced oxidative stress and mitochondrial dysfunction, suppressing premature senescence by inhibiting activation of the p53–p21 and p16^-INK4A^–pRB pathways.

## Introduction

Synephrine is a primary protoalkaloid, that is found mainly in the peels of various citrus fruits, such as bitter oranges. In China, synephrine is used as an herbal medicine to treat digestive problems [1].

Synephrine has three different isometric forms: ortho (*o*-), meta (*m*-), and para (*p*-) forms [2]. Among these three isometric forms, *p*-synephrine has been extensively investigated for its biological effects.

*p*-Synephrine stimulates glucose consumption and lactic acid production via AMP-activated protein kinase (AMPK) activity in L6 skeletal muscle cells [3]. Synephrine also activates the β3 adrenergic receptor to induce lipolysis [4, 5]. Therefore, synephrine is widely used as a dietary supplement for weight loss.

*p*-Synephrine also exerts anti-inflammatory effects. It inhibits the production of proinflammatory cytokines and nitric oxide in lipopolysaccharide -stimulated RAW264.7 cells via downregulation of the p38 MAPK and nuclear factor-κB signaling pathways. Additionally, *p*-synephrine alleviates systemic inflammatory response syndrome in mice [6].

*p*-Synephrine significantly decreases reactive oxygen species (ROS) generation and myeloperoxidase activity and storengthens superoxide dismutase activity in mice with lipopolysaccharide-induced acute lung injury [7].

Cellular senescence is induced by various stresses and plays important roles in aging and the onset of various age-related diseases, including cancer. WI-38 cells are human fetal lung-derived fibroblasts established in 1962 by Dr. Hayflick. Hayflick first demonstrated that normal human somatic cells inevitably undergo irreversible cell cycle arrest when passaged repeatedly in culture dishes [8]. Stress-induced premature senescence (SIPS) also induces cell cycle arrest by leading to various types of cellular stress, such as DNA damage and oxidative stress, without telomere shortening [9]. Lowers et al. revealed that oncogenic RAS expression induces premature cellular senescence and that induction of cellular senescence serves as a tumor suppression mechanism [10].

Senescent cells secrete various factors, such as inflammatory cytokines, chemokines, and extracellular matrix-degrading enzymes, and these cells exert inflammatory and carcinogenic effects, resulting in a senescence-associated secretory phenotype (SASP) [9, 11].

Senescent and cancerous cells are resistant to apoptosis, and Bcl-2 family antiapoptotic proteins are highly expressed in these cells. ABT-263 (Navitoclax) and ABT-737, which are Bcl-2 family inhibitors, selectively remove senescent cells and restore the activity of various stem cells [12–14]. Several senolytics, such as ABT-263 and ABT-737, which are used in anticancer drugs to selectively remove senescent cells, have been recently developed.

The free-radical theory of senescence was proposed by Harman in 1956 [15]. Antioxidants and antioxidant enzymes extend the lifespan of animals. For example, mutant mice lacking manganese superoxide dismutase, which acts as a primary defense against mitochondrial superoxide, survive for only a couple of weeks after birth and exhibit cardiomyopathy, metabolic acidosis, and central nervous system degeneration due to mitochondrial dysfunction [16, 17]. The average lifespan of mice deficient in vitamin C is 6 months, which is only approximately one-fourth of their healthy lifespan [18]. The accumulation of senescent cells contributes to arteriosclerosis [19], diabetes [20], and a shortened lifespan [21]. The findings of these reports indicate that antioxidants and antioxidant enzymes possibly attenuate ROS-induced oxidative stress by scavenging free radicals, thereby protecting against oxidation-related diseases and aging-related processes, including cellular senescence.

The biological effects of synephrine have been reported in many studies. However, the antioxidant effects of synephrine on cultured cells remain unclear. In this study, we aimed to assess the antioxidant activity of synephrine against H_2_O_2_-induced premature senescence using WI-38 cells.

## Materials and Methods

### Cell Culture

WI-38 cells (human fetal lung-derived fibroblasts) were obtained from the Riken Cell Bank (Riken BioResource Research Center [Riken BRC], Ibaraki, Japan) and cultured in Dulbecco’s modified Eagle’s medium (Sigma-Aldrich, St. Louis, MO) supplemented with 10% fetal bovine serum (Biosource International, Camarillo, CA) and 1% penicillin–streptomycin at 37 °C and 5% CO_2_. WI-38 cells at approximately 20–25 population doubling (PD) were used to avoid the effects of replicative senescence. The cells (5 × 10^4^ or 1 × 10^5^ cells/mL) were seeded and incubated for 24 h. After incubation, the culture medium was removed, and the cells were treated with synephrine for 3 h. Then, the cells were treated with 150 µM H_2_O_2_ for 1 h to induce SIPS. After H_2_O_2_ treatment, the medium containing H_2_O_2_ was replaced with fresh medium for use in subsequent experiments. The medium was replaced every 2–3 d. Synephrine was purchased from Tokyo Chemical Industry Co. Ltd. Japan and H_2_O_2_ from FUJIFILM Wako Pure Chemical Corporation (Osaka, Japan). The purity of synephrine was >98%, as determined by HPLC, titration analyses.

### Cell Viability

Next, the cytotoxicities of synephrine and H_2_O_2_ were evaluated using a water-soluble tetrazolium salt assay (Cell Counting Kit-8; Dojindo, Kumamoto, Japan). After synephrine (0, 25, 50, and 100 µM) treatment for 3 h at 37 °C and 5% CO_2_ and H_2_O_2_ (0, 50, 100, and 150 µM) treatment for 1 h at 37 °C and 5% CO_2_, water-soluble tetrazolium salt assays were performed according to the manufacturer’s instructions.

### Evaluation of Senescence-Associated β-Galactosidase (SA-β-Gal) Activity and Senescence-Associated Heterochromatin Foci (SAHF) during H_2_O_2_-Induced Premature Senescence

SA-β-gal activity was measured using the Cellular Senescence Plate Assay kit-SPiDER-βGal (SG05; Dojindo). Five days after treatment, the cells were collected, and 100 µL of cell lysate was seeded in each well of a 96-well black plate (Sumitomo Bakelite Co., Tokyo, Japan) for 6 h. After the supernatant was removed, an SA-β-gal assay was performed according to manufacturer’s protocol. The fluorescence intensity was measured at an excitation wavelength of 535 nm and an emission wavelength of 580 nm with a plate reader (Infinite M200; TECAN Group Ltd., Männedorf, Switzerland). The number of cultured cells was adjusted with a Cell Count Normalization Kit (C544; Dojindo). Then, 4′,6-diamidino-2-phenylindole (DAPI) staining was performed to visualize SAHF. The cells were fixed with 4% formaldehyde for 30 min at room temperature and washed three times with phosphate-buffered saline (PBS). Then, the fixed cells were stained with DAPI (1 µg/mL) for 20 min and washed. Finally, SAHF were observed under a microscope (BZ X-700; Keyence, Osaka, Japan), setting the magnification ratio to 40× and the exposure time to 1/6 s.

### Electron Spin Resonance (ESR) Spectroscopy

The radical scavenging activity of synephrine was evaluated via ESR spectroscopy. Hydroxyl radicals were generated via the Fenton reaction with 200 μM H_2_O_2_ and 20 μM FeSO_4_, and superoxide anion radicals were generated with 2.76 mM hypoxanthine, 138 U/L xanthine oxidase and 109 μM diethylenetriamine-N,N,N′,N″,N″-pentaacetic acid. These radicals were detected using a spin-trapping method with 5,5-dimethyl-1-pyrroline-N-oxide (LABOTEC Co., Ltd., Tokyo, Japan). ESR spectra in the presence or absence of 10 and 1000 μM synephrine were acquired with a JEOL X-band spectrometer (JES-RE1X; JEOL Ltd., Tokyo, Japan) at 25 °C. The ESR measurements conditions were as follows: microwave frequency, 9.40 GHz; microwave power, 5 mW; center field, 336.5 mT for the hydroxyl radical and 335.5 mT for the superoxide radical; sweep width, 15 mT; sweep time, 1 min; modulation amplitude, 0.1 mT for the hydroxyl radical and 0.2 mT for the superoxide radical; and time constant, 0.1 s. EPR data acquisition was performed using the WIN-RAD ESR Data Analyzer System (Radical Research, Inc., Tokyo, Japan). Ascorbic acid, a strong antioxidant, was used as the positive control to scavenge radicals.

### Measurement of ROS Levels

ROS levels were determined using the fluorescent dye hydroxyphenyl fluorescein (HPF; Daiichi Pure Chemicals Co., Tokyo, Japan). The cells were cultured on 35-mm glass-bottom dishes (MatTek Corp. Ashland, MA, USA), incubated at 37 °C and 5% CO_2_ for 24 h, and treated with synephrine and H_2_O_2_. After the medium was replaced with fresh medium, the cells were incubated for 2 h. The medium was replaced with modified Hanks’ balanced salt solution (MSF solution) containing 10.0 mM HEPES, 1.0 mM MgCl_2_, 2 mM CaCl_2_, and 2.7 mM glucose adjusted to pH 7.3 ± 0.05 and incubated at 37 °C for 15 min in the dark. Bioimages of HPF fluorescence were obtained with a CSU-10 confocal laser scanning system (Yokogawa Electric Co., Tokyo, Japan) coupled to an IX90 inverted microscope with a UPlanAPO 20× objective lens (Olympus Optical Co., Tokyo, Japan) and a C5810-01 color chilled CCD camera (Hamamatsu Photonics, Hamamatsu, Japan).

### Lipid Peroxidation and Oxidative DNA Damage Analysis via Immunostaining

Immunofluorescence staining was performed to determine the levels of 4-hydroxynonenal (4-HNE). The cells were fixed with 1 mL 4% formaldehyde/PBS at room temperature for 30 min and washed three times with PBS, and the cell membranes were permeabilized with 95% ethanol and 5% acetic acid at room temperature for 10 min. After being washed five times with PBS, the cells were incubated with blocking serum (1% bovine serum albumin/PBS) at room temperature for 30 min. Immunoblotting was performed by incubating the cells with anti-4-HNE and anti-8-hydroxydeoxyguanosine (8-OHdG) monoclonal antibodies (JaICA, Shizuoka, Japan) at 4 °C overnight, followed by incubation with the Alexa Fluor 488 goat anti-mouse IgG (H + L) conjugate (1/200 dilution; Molecular Probes, Eugene, OR, USA) for 1 h at room temperature. The laser beam intensity, amplifier gain, and analysis method were the same as those used for the HPF staining.

### Measurement of the Mitochondrial Membrane Potential

The fluorescent probe 5,5′,6,6′ tetrachloro-1,1′3,3′ tetraethylbenzimidazolcarbocyanine iodide (JC-1; Thermo Fisher Scientific, Tokyo, Japan) was used to assess the mitochondrial membrane potential. After 72 h of H_2_O_2_ treatment, the medium was replaced with MSF solution containing 5 µM JC-1, and the cells were incubated at 37 °C for 30 min in the dark. After incubation, green and red fluorescence was observed at 488 and 568 nm, respectively, using a double-window barrier filter. Changes in the mitochondrial membrane potential were evaluated using the red/green fluorescence ratio.

### ATP Quantification

Five days after the H_2_O_2_ treatment, an ATP luminescence assay kit (A550; Dojindo) was used to evaluate ATP production. The cells (5 × 10^4^ cells/mL) were seeded in Dulbecco’s modified Eagle’s medium containing 10% fetal bovine serum in a 60-mm dish. After treatment, the medium was replaced with a fresh medium, and the cells were cultured for five days at 37 °C in a 5% CO_2_ incubator. Finally, ATP levels were measured according to manufacturer’s instructions.

### Mitophagy Detection

Five days after the H_2_O_2_ treatment, mitophagy was detected with the Mitophagy Detection Kit (MD01; Dojindo). The cells were cultured on 35-mm glass-bottom dishes (MatTek Corp.). After the medium was removed and cells were washed twice with Hank’s HEPES buffer, 100 nM Mtphagy Dye working solution was added, and the cells were incubated at 37 °C for 30 min. After the cells were washed twice with Hank’s HEPES buffer and mixing, medium containing the mitophagy-inducing agent carbonyl cyanide-*p*-trifluoromethoxyphenylhydrazone (Funakoshi, Tokyo, Japan) was added, and mitophagy localization was analyzed using a fluorescence microscope. Furthermore, 1 µM Lyso dye working solution was added to the dishes, and the cells were incubated at 37 °C for 30 min. After the supernatants were discarded and the cells were washed twice with Hank’s HEPES buffer, mitophagy was observed using a confocal laser microscope. The absorbance of the Mtphagy Dye and Lyso dye at 561 and 488 nm, respectively, were determined using a double-window barrier filter.

### Western Blotting Analysis

Briefly, harvested cells were collected and centrifuged at 1500 × *g* for 5 min at 4 °C. The pellet was resuspended in a radioimmunoprecipitation assay buffer and dissolved in an ultrasonic cell disruptor. The suspension was centrifuged at 10,000× *g* for 10 min at 4 °C, and the supernatant was collected. The protein concentration was measured using the bicinchoninic acid (BCA) protein assay reagent (Pierce, Rockford, IL, USA). Whole-cell lysates were dissolved in western blotting sample buffer. Then, the cell lysates (20 µg protein) were heated at 95 °C for 5 min and subjected to sodium dodecyl sulfate-polyacrylamide gel electrophoresis on 10–15% gels for each cell cycle arrest-related protein. The proteins were electrophoretically transferred to polyvinylidene difluoride membranes (Merck Millipore, Burlington, MA, USA) at 2 mA/cm^2^ for 60 min. After blocking with 5% (w/v) skim milk solution for 60 min, the membranes were incubated with anti-p16^-INK4A^ polyclonal (1:1000; 10883-1-AP; Cosmo Bio Co, Ltd., Tokyo, Japan), anti-p21 polyclonal (1:1000; 28248-1-AP; Cosmo Bio Co, Ltd.), and anti-p53 (1:1000; 2524S, Cell Signaling Technology, Danvers, MA, USA) primary antibodies at 4 °C overnight, followed by incubation with horse anti-mouse IgG-horseradish peroxidase (HRP; 1:5000; 7076S; Cell Signaling Technology) and goat anti-rabbit IgG-HRP (1:5000; 7074S; Cell Signaling Technology) secondary antibodies for 3–4 h at room temperature. After the Lumina Forte Western HRP substrate (Merck Millipore) was applied, luminescence was detected using FluorChemFC2 (Alpha Innotech Co., San Leandro, CA).

### Statistical Analyses

Statistical analyses were conducted using the Scheffé’s test with the IBM SPSS Statistics software (International Business Machines Corp., NY). The threshold for statistical significance was set as *P* < 0.05.

## Results

### Cell Viability

**Fig. 1:**
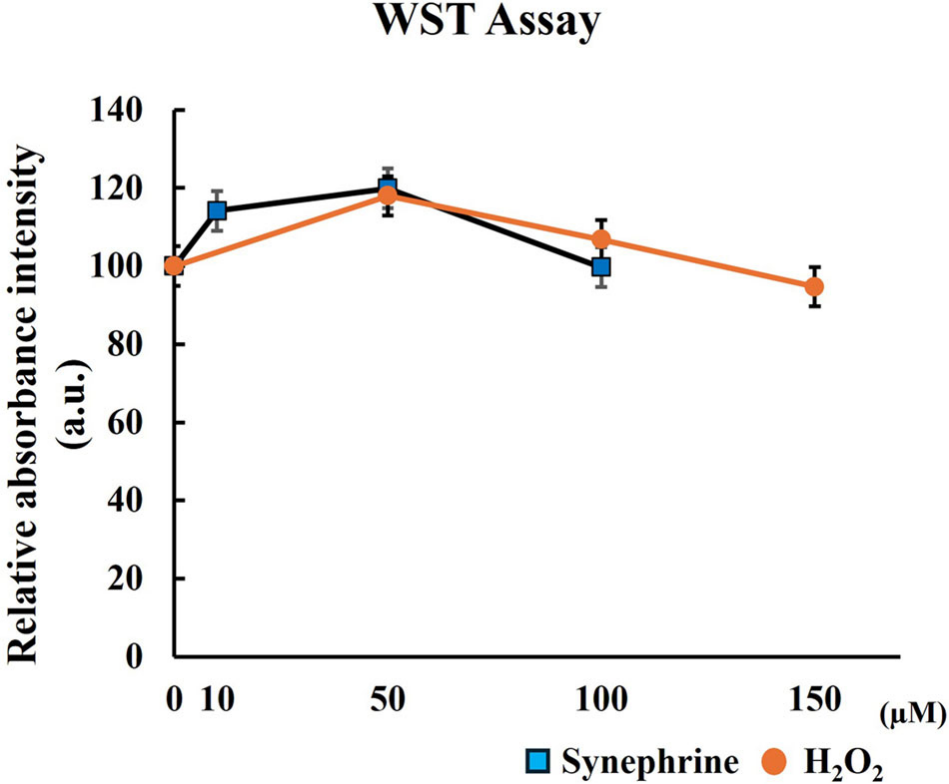
Viability of synephrine-treated and H_2_O_2_-induced cells determined via water-soluble tetrazolium salt (WST) assay. The viability of all the tested WI-38 cells was greater than 90%

Here, 90% cell viability was observed after 3 h treatment with 100 µM synephrine; therefore, concentrations of synephrine up to 50 µM were used in the following experiments. In contrast, cells treated with 150 µM H_2_O_2_ for 1 h exhibited over 94% cell viability (Fig. 1). These results confirmed that the doses of synephrine and H_2_O_2_ used here were non-toxic to WI-38 cells.

### Changes in Cell Morphology and Cellular Senescence Index

Next, we evaluated the changes in cell morphology and the senescence index five days after H_2_O_2_ treatment.

**Fig. 2:**
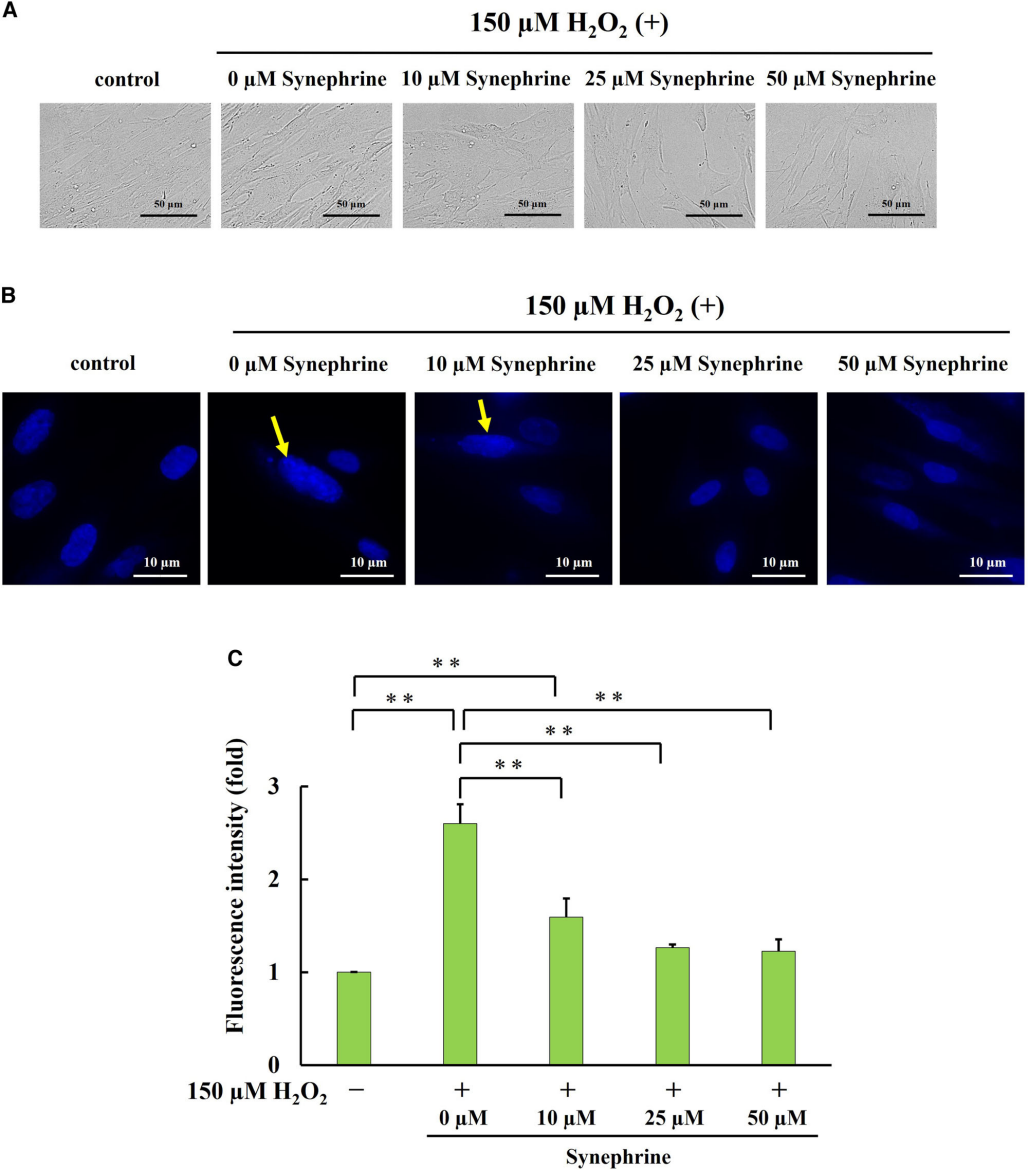
H_2_O_2_-induced premature senescence phenotypes. **A** Cell morphology changes. Representative images of each group were obtained via bright-field microscopy (magnification ratio: ×40). **B** Detection of senescence-associated heterochromatin foci (SAHF) via 4′,6-diamidino-2-phenylindole (DAPI) staining (magnification ratio: ×40). Arrows indicate SAHF. **C** Senescence-associated β-galactosidase (SA-β-gal) activity, a cellular senescence biomarker, was measured. Bar: Mean ± S.D.; n = 3; ** *P* < 0.01 according to Scheffé’s test

Morphological changes in H_2_O_2_-induced senescent cells were observed using bright-field microscopy. Cells without H_2_O_2_ exposure were thin with small nuclei, whereas the shape and size of H_2_O_2_-treated cells were greater than those of untreated cells and were lower than those of synephrine-treated cells (Fig. 2A). These results indicate that synephrine reverses the H_2_O_2_-induced morphological changes.

Various stresses, including oxidative stress, induce double-strand breaks and DNA damage, triggering the DNA damage response; activation of the DNA damage response is essential for SAHF expression [22]. Here, DAPI staining revealed SAHF expression in H_2_O_2_-treated cells. However, DAPI-stained DNA foci were almost undetectable in the synephrine-treated cells (Fig. 2B). SA-β-gal activity, which is widely used as a biomarker of cellular senescence, was measured. Compared with the control treatment, H_2_O_2_ treatment increased SA-β-gal activity. However, synephrine protected against H_2_O_2_-induced premature senescence by significantly decreasing SA-β-gal activity (Fig. 2C).

### Evaluation of the ROS Scavenging Activity of Synephrine using ESR Spectroscopy

**Fig. 3:**
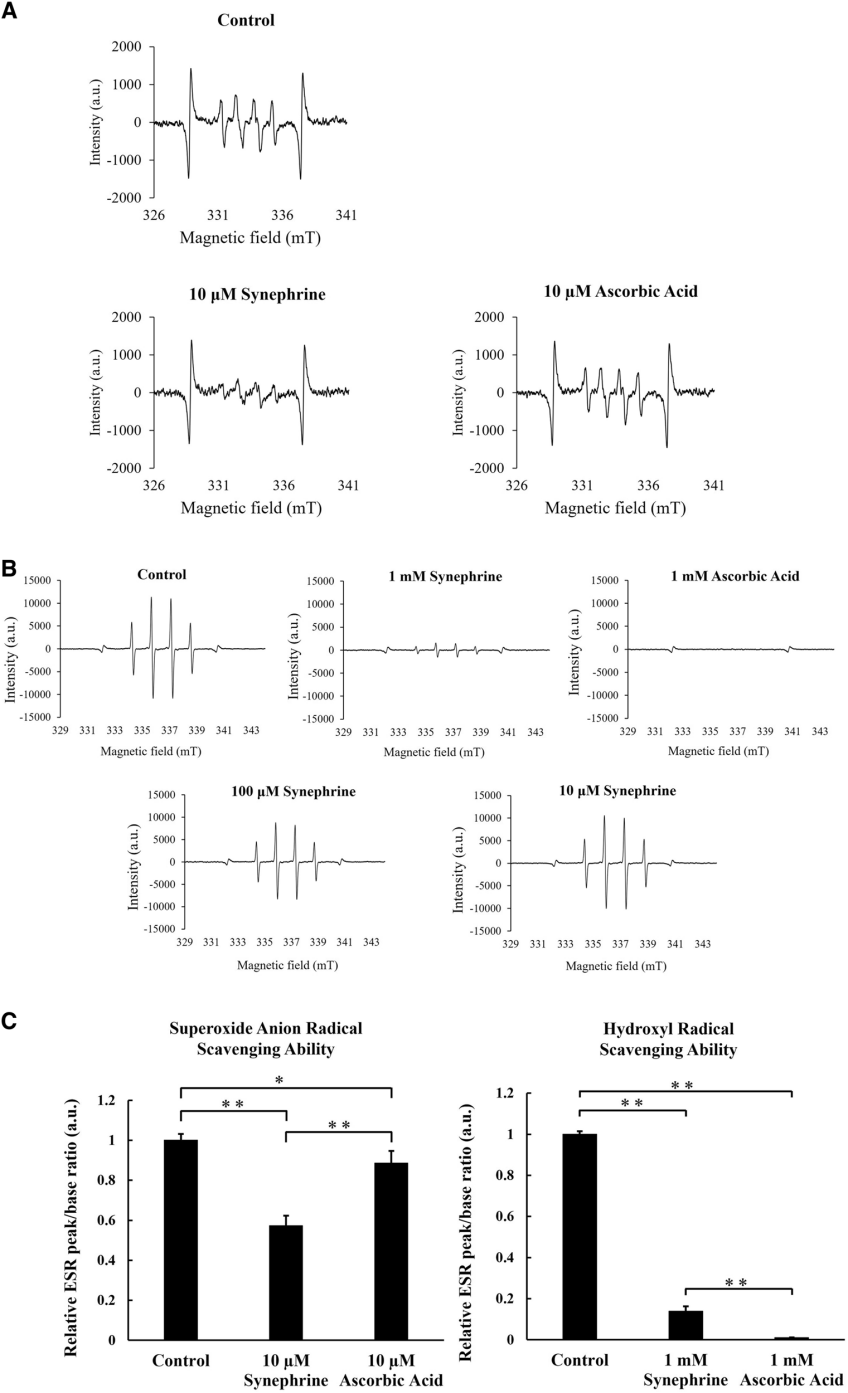
Electron spin resonance (ESR) spectra were recorded using 5,5-dimethyl-1-pyrroline-N-oxide (DMPO) to investigate the radical scavenging activity of synephrine. **A** Amplitudes of negative control, 10 µM synephrine, and 10 µM ascorbic acid for superoxide anion radical scavenging activity. **B** Amplitudes of the negative control; 1 mM ascorbic acid, and 10 µM; 100 µM, and 1 mM synephrine for hydroxy radical scavenging activity. **C** Relative ESR peak/base ratios of the superoxide anion and hydroxyl radicals. Bar of the superoxide anion radical scavenging test: Mean ± standard deviation (S.D.); n = 4; *P < 0.05 and **P < 0.01 according to Scheffé’s test. Bar of hydroxy radical scavenging test: Mean ± S.D.; n = 3; ** *P* < 0.01 according to Scheffé’s test

Evaluation of the superoxide anion radical scavenging activity revealed that the amplitude of 10 μM synephrine was lower than that of 10 μM ascorbic acid. Moreover, the peak-to-base ratio of 10 μM synephrine was significantly lower than that of 10 μM ascorbic acid and lower than that observed without the addition of any reagent (Fig. 3A, C). Therefore, the superoxide anion radical scavenging activity of synephrine was greater than that of ascorbic acid, a known antioxidant. Analysis of hydroxyl radical scavenging activity revealed almost no decrease in amplitude with 10 and 100 μM synephrine but a sufficient decrease in amplitude with 1 mM synephrine. Additionally, the peak-to-base ratio of 1 mM synephrine was significantly greater than that of 1 mM ascorbic acid but significantly lower than that observed without the addition of any reagent (Fig. 3B, C). Therefore, synephrine can scavenge ROS, but the amount of oxygen eliminated depends on the type of ROS.

### Analysis of ROS Generation, Lipid Peroxidation, and DNA Damage

To determine the effects of synephrine, the cells were treated with synephrine (10, 25, and 50 µM) for 3 h before exposure to 150 µM H_2_O_2_ for 1 h. Intracellular ROS levels, lipid peroxidation, and oxidative DNA damage were evaluated 2 h after treatment with H_2_O_2_. To quantitatively evaluate the fluorescence intensity, more than 100 cells were counted in each image to calculate the average fluorescence intensity/cell.

**Fig. 4:**
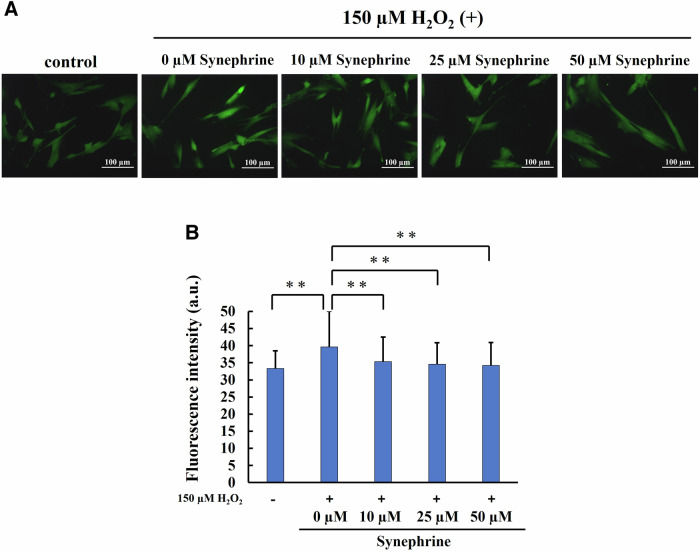
Effect of synephrine on reactive oxygen species (ROS) generation in H_2_O_2_-treated WI-38 cells. Intracellular ROS levels were determined via the use of a hydroxyphenyl fluorescein (HPF) fluorescence probe. **A** Representative HPF images (magnification ratio: ×20). **B** Fluorescence intensity determined from the representative HPF images in (A). Quantitative assessment of fluorescence intensity over 100 cells. Bar: Mean ± S.D.; n = 3; **P < 0.01 according to Scheffé’s test

HPF reacts with highly ROS, such as hydroxyl radicals and peroxynitrite, to generates green fluorescence at excitation wavelengths ranging from approximately 490–540 nm. Here, the frequency of ROS generation was greater in H_2_O_2_-treated cells than in untreated cells. Compared with that in H_2_O_2_-treated cells, the fluorescence intensity was significantly lower in synephrine (10, 25, and 50 µM)-treated cells (Fig. 4A). The quantitative results confirmed this observation, indicating that synephrine significantly decreased the ROS levels induced by H_2_O_2_ in a dose-dependent manner (Fig. 4B).

**Fig. 5:**
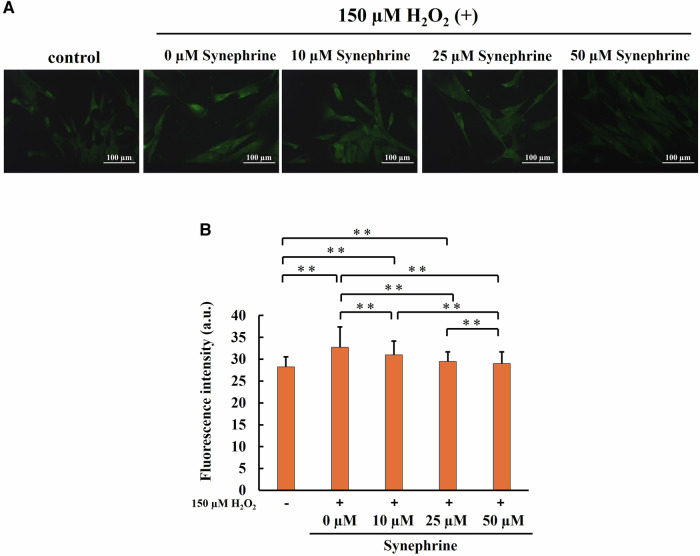
Effect of synephrine on lipid peroxidation in H_2_O_2_-treated WI-38 cells determined using an anti-4-hydroxynonenal (4-HNE) antibody. **A** Representative images of 4-HNE staining in each group (magnification ratio: ×20). **B** Quantitative assessment of fluorescence intensity in 4-HNE-stained cells. Bar: Mean ± S.D.; n = 3; **P < 0.01 according to Scheffé’s test

To determine whether changes in intracellular ROS generation were accompanied by changes in lipid peroxidation, 4-HNE levels were determined via immunocytochemical staining. The fluorescence intensity in the H_2_O_2_-treated cells was significantly higher than that in the control cells but significantly reduced after treatment with 10–50 μM synephrine in a dose-dependent manner (Fig. 5A, B).

**Fig. 6:**
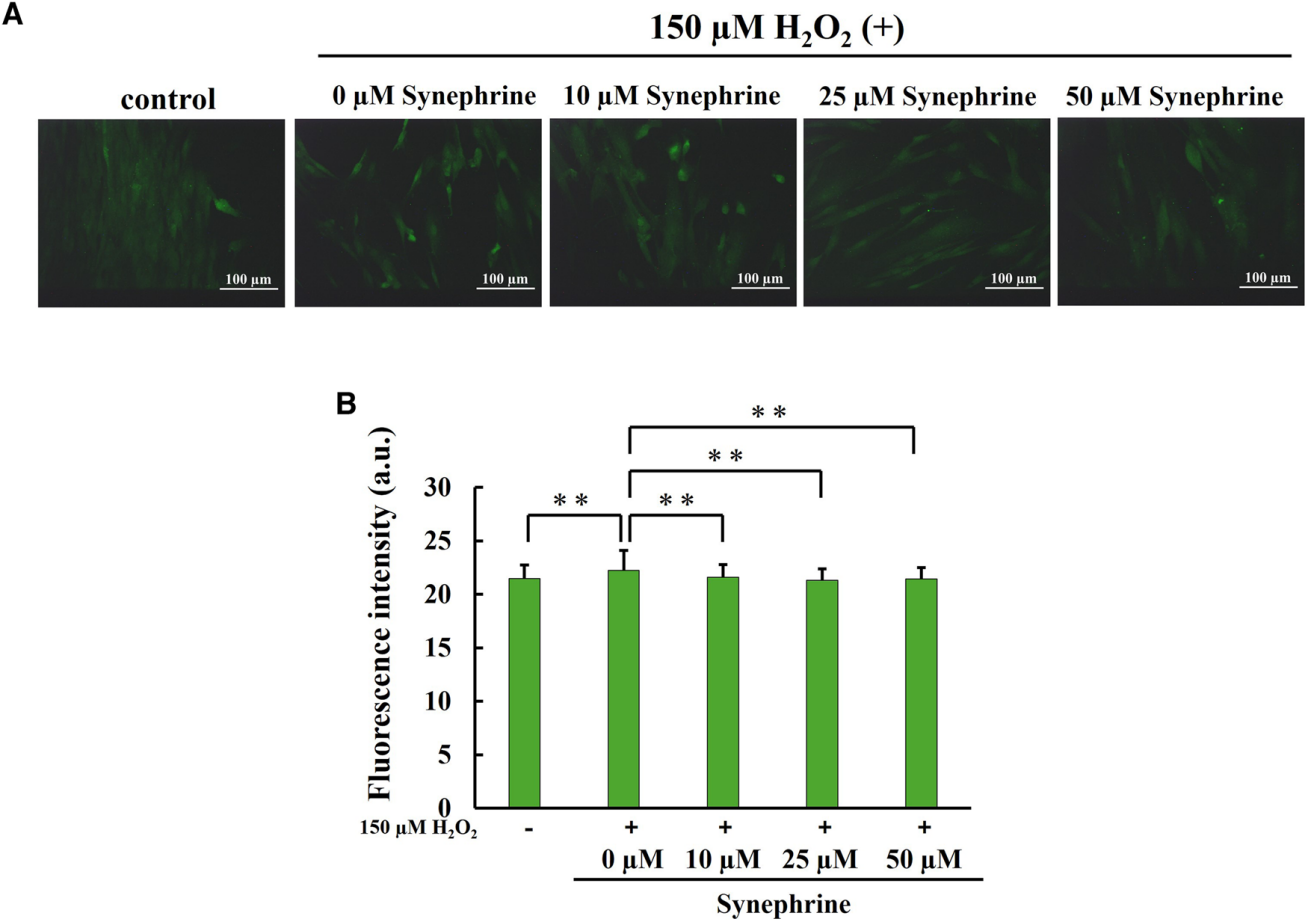
Effect of synephrine on oxidative DNA damage in H_2_O_2_-treated WI-38 cells. **A** Representative images of anti-8-hydroxydeoxyguanosine (8-OHdG) antibody immunostaining in each group (magnification ratio: ×20). **B** Quantitative assessment of fluorescence intensity in 8-OHdG-stained cells. Bar: Mean ± S.D.; n = 3; **P < 0.01 according to Scheffé’s test

Hydroxylation of C8 on deoxyguanosine (dG), an easily oxidized DNA base, results in the formation of 8-OHdG [23]. Therefore, 8-OHdG is widely used as a biomarker for oxidative DNA damage. Similar to the results of HPF staining and lipid peroxidation analysis, increased fluorescence intensity of H_2_O_2_-treated cells was significantly decreased by synephrine treatment (Fig. 6A, B).

These results suggest that synephrine inhibits H_2_O_2_-induced oxidative stress.

### Evaluation of the Mitochondrial Function

To clarify whether synephrine influences mitochondrial dysfunction, cells were pretreated with or without synephrine for 3 h and stimulated with 150 µM H_2_O_2_ for 1 h.

We examined the effects of synephrine on mitochondrial membrane potential and ATP production during H_2_O_2_-induced premature senescence.

JC-1 detects differences in the mitochondrial membrane potential as differences in fluorescence intensity. JC-1 is aggregated and emits red fluorescence in the high-potential region, but it is distributed and emits green fluorescence in the low-potential region.

**Fig. 7:**
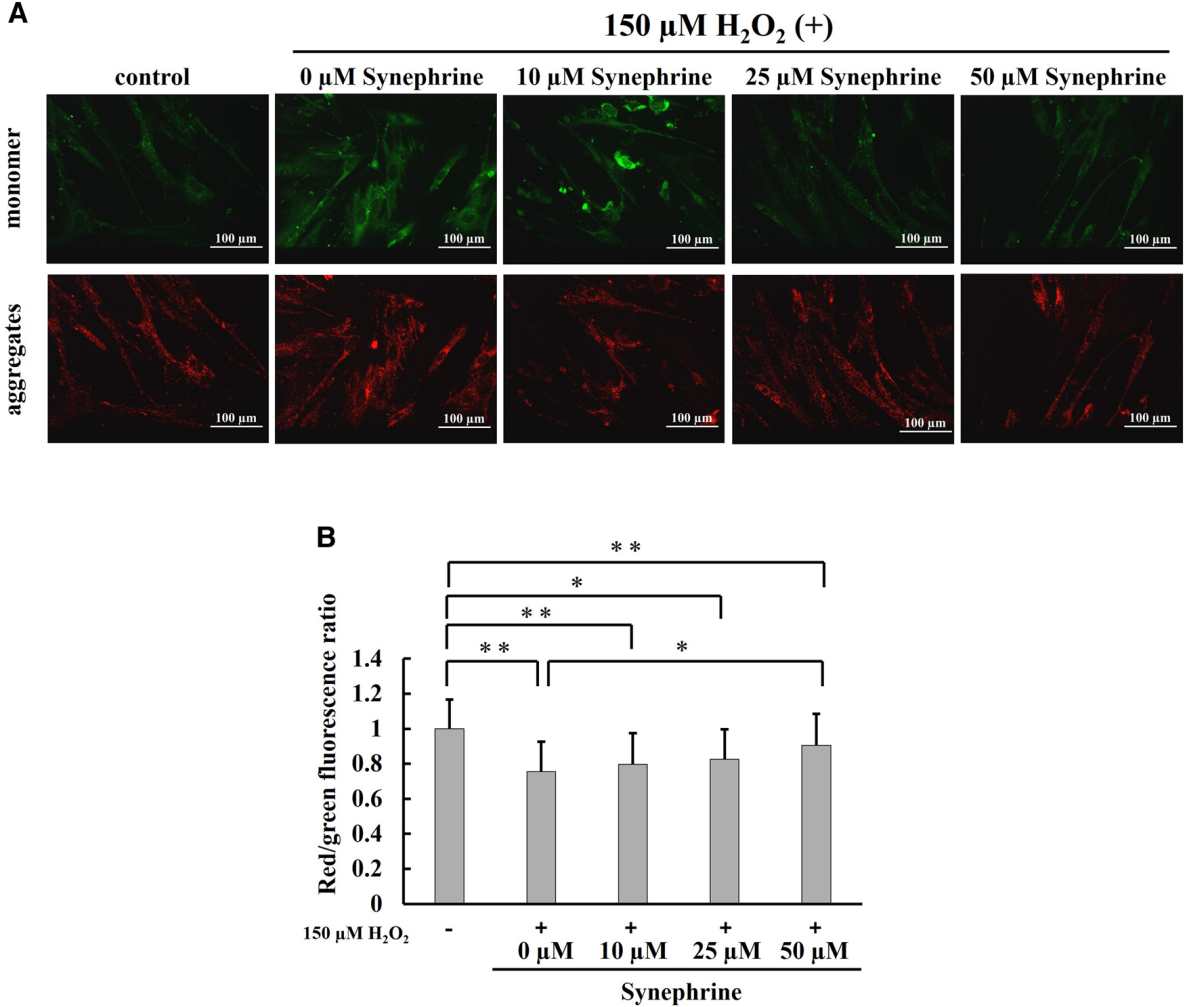
Mitochondrial membrane potential determined using 5,5′,6,6′ tetrachloro-1,1′3,3′ tetraethylbenzimidazolcarbocyanine iodide (JC-1) and confocal microscopy. **A** Representative images of JC-1 staining in each group (magnification ratio: ×20). JC-1 is aggregated and emits red fluorescence in regions with high mitochondrial membrane potential. In contrast, JC-1 is distributed and emits green fluorescence in regions with low mitochondrial membrane potential. **B** JC-1 red/green fluorescence ratio indicating the mitochondrial membrane potential. Bar: Mean ± S.D.; n = 3; *P < 0.05 and **P < 0.01 according to Scheffé’s test

At 72 h after H_2_O_2_ treatment, green and red fluorescence was observed using a JC-I fluorescence probe. Changes in mitochondrial membrane potential were evaluated using the red/green fluorescence ratio. Red/green fluorescence intensity ratio was significantly reduced in the H_2_O_2_-treated cells compared to that in the control cells; however, this ratio was significantly restored only in the 50 μM synephrine-treated cells (Fig. 7A, B).

**Fig. 8:**
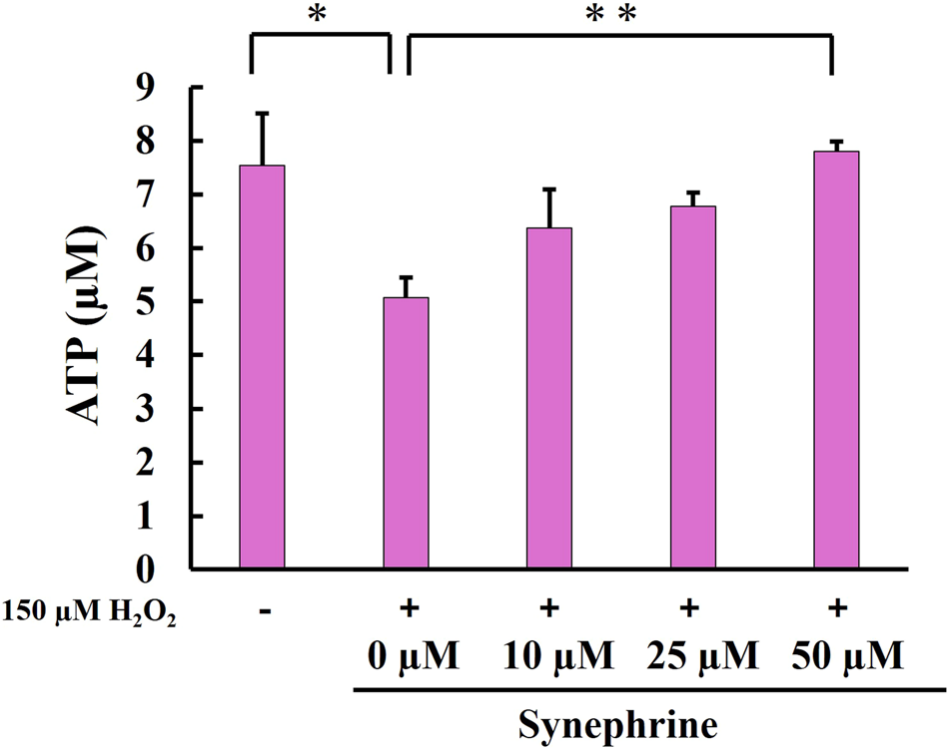
Quantification of intracellular ATP levels via a luciferase luminescence assay. ATP concentration in each group was measured using a calibration curve plotted using the 0–10 µmol/L ATP standard. Bar: Mean ± S.D.; n = 3; *P < 0.05 and **P < 0.01 according to Scheffé’s test

ATP production was evaluated five days after H_2_O_2_ treatment using an ATP assay kit. ATP levels were significantly lower in the H_2_O_2_-treated cells than in the control cells but significantly restored in 50 μM synephrine-treated cells (Fig. 8).

### Mitophagy Detection

Mitophagy is a selective process that removes damaged mitochondria and controls mitochondrial quality. We verified whether the protective effects of synephrine against H_2_O_2_-induced premature senescence are related to increased mitophagy.

**Fig. 9:**
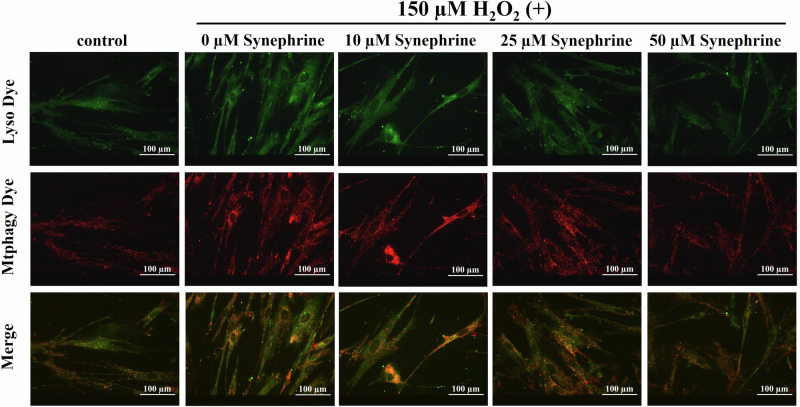
Mitophagy detection via Mtphagy and Lyso (lysosome-selective) dyes. Merged double images of Mtphagy and Lyso dyes fluorescence were constructed to detect mitophagy. Mitophagy was detected in the yellow regions of the merged images. Representative images of mitophagy are shown in Fig. 8 (magnification ratio: ×20)

Five days after H_2_O_2_ treatment, cells were stimulated with 100 µM carbonyl cyanide-*p*-trifluoromethoxyphenylhydrazone for 6 h to observe mitophagy. Next, we used a Mtphagy Dye, which detects mitophagy by costaining with Lyso Dye. Mtphagy Dye permeates the cell membrane and accumulates in the mitochondria, where it is immobilized via chemical bonding. In this state, fluorescence intensity is low. When mitophagy is induced and mitochondria fuse with lysosomes, fluorescence intensity of the Mtphagy Dye increases. As shown in Fig. 9, mitophagy level was increased in the H_2_O_2_-treated but decreased in the 50 μM synephrine-treated WI-38 cells.

### Expression of Cellular Senescence-Related Proteins

Senescent cells are characterized by irreversible growth arrest in the G1 phase. Expression levels of cell cycle-inhibiting genes *p16*^*INK4a*^ and *p53* and their target transposable factor p21^CIP1^ are elevated in senescent cells [24]. Therefore, expression levels of p16^-INK4A^, p21, and p53 five days after H_2_O_2_ treatment were examined via western blotting analysis.

**Fig. 10:**
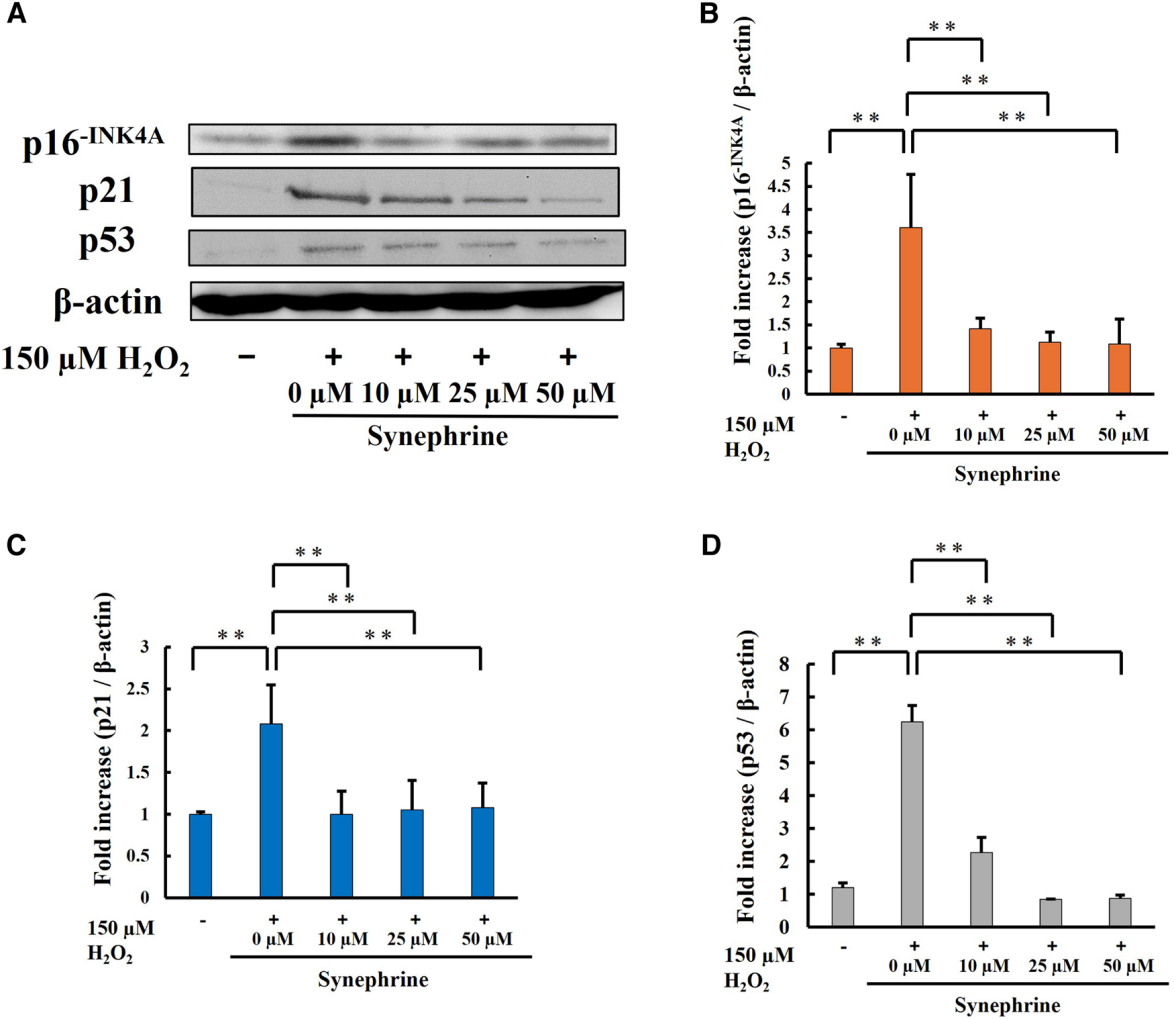
Expression levels of cell cycle arrest markers. **A** Expression levels of cell cycle arrest markers were determined via western blot analysis. Total cell lysates (20 μg/lane) from each sample were subjected to sodium dodecyl sulfate-polyacrylamide gel electrophoresis (SDS-PAGE) on 10–15% gels for resolution of p53, p21, and p16^-INK4A^. β-Actin was used as a loading control. Quantitative assessment of band intensities for (**B**) p16^-INK4A^, (**C**) p21 and (**D**) p53. Bar: Mean ± S.D.; n = 3; **P < 0.01 according to Scheffé’s test

Levels of p53 and p21 were higher in the H_2_O_2_-treated cells than in the control cells, but these levels were downregulated by synephrine treatment. Notably, p16^-INK4A^ levels showed similar changes as p53 and p21 levels (Fig. 10A). Figure 10B–D shows the quantitative assessment of the intensity of each band.

## Discussion

In this study, we aimed to elucidate the effects of synephrine on SIPS and examine its roles in mitochondrial functions onWI-38 cells.

WI-38 cells are normal human-derived cells, and Hayflick first demonstrated that these normal human somatic cells undergo irreversible cell cycle arrest when repeatedly passaged in culture dishes. Therefore, WI-38 has been used in many cellular senescence studies including SIPS.

Cellular senescence induced by various stressors is known as SIPS. Ionizing radiation [25, 26], ultraviolet radiation [27], H_2_O_2_ [27–29], and pharmacological agents, such as doxorubicine and *tert*-butylhydroperoxide [30, 31], are widely used as inducers of SIPS.

First, we examined the cytotoxic effects of synephrine and H_2_O_2_ on WI-38 cells. Synephrine and caffeine alone were not cytotoxic to HepG2 cells; however, the combination of synephrine and caffeine led to DNA damage and apoptosis.

However, they only observed cytotoxicity of synephrine at concentrations of 0.03–30 µM for 24 h in HepG2 cell [32]. Treatment with concentrations up to 50 µM synephrine treatment for 3 h did not have a cytotoxic effect on WI-38 cells (Fig. 1). The cytotoxicity of synephrine in cultured cells is possibly affected by the cell type and treatment conditions. Notably, no cytotoxicity resulting from 150 µM H_2_O_2_ treatment for 1 h was observed in WI-38 cells. Additionally, apoptosis was not observed after 0, 25, 50, and 100 µM synephrine (data not shown).

As the characteristic phenotypes of cellular senescence, morphological changes in cells, SAHF, SASP, increased SA-β-gal activity are observed in senescence cells [33–35]. Therefore, these are widely used as markers of cellular senescence. When cellular senescence is induced by various stress, Nectin-4 expression is induced in a p53-dependent manner, and Nectin-4 transmits signals through Src family kinase, PI3 kinase, and Rac causing an increase in cell size. [36] In our results, the morphological changes of in the calls cells including large and flat shapes, were observed in H_2_O_2_ treated cells, but in cells treated with synephrine, the morphological changes were found to be reduced (Fig. 2A). Chromatin structure changes due to DNA damage, and the tight heterochromatin region increases. A specific heterochromatic structure called SAPH was observed, whereas some articles reported that SAHF is not observed in senescent cells [37]. Although SAHF is a characteristic phenotypes of cellular senescence, it is not essential. In our study, H_2_O_2_ treatment induced SAHF formation in senescent cells (Fig. 2B). Furthermore, synephrine treatment reduced SAHF levels in senescent cells. Our results suggest that synephrine reduces oxidative DNA damage in H_2_O_2_ induced cells, resulting in the suppression of SAHF. The activation of SA-β-gal is associated with changes in the metabolism and function of senescence cells, and its activity is known to increase due to cell cycle arrest, DNA damage, and the accumulation of oxidative stress. In our results, the SA-β-gal activity in H_2_O_2_ treated cells was found to be significantly increased and decreased in a concentration-dependent manner with synephrine treatment (Fig. 2C). These results suggested that synephrine suppressed H_2_O_2_-induced premature senescence. We hypothesized that synephrine, which exhibits antioxidant activity, suppresses oxidative stress, thereby preventing H_2_O_2_-induced premature senescence of WI-38 cells.

Synephrine is a protoalkaloid present in many dietary supplements. Natural sources of synephrine are the peels of *Citrus aurantium* (CA; also known as bitter orange or Seville orange sour orange) and other citrus species, such as “Zhi Shi,” “Kijitsu,” and “Satsuma orange” [38–40]. Watson et al. reported that synephrine is present in humans and is considered a trace amine because of its low plasma levels [41].

Synephrine exerts anti-inflammatory effects and induces vasoconstriction, bronchodilation, lipolysis, and activation of the cardiovascular and central nervous systems [42]. Ribeiroa DL et al., reported that 6 h treatment with 200 µM synephrine resulted in the overproduction of intracellular ROS in HepG2 cells [43]. Wang YL et al. reported that *p*-synephrine inhibits oxidative stress by suppressing the nuclear factor-κB and mitogen-activated protein kinase pathways, thereby alleviating alloxan-induced diabetes in mice [44]. However, whether synephrine acts as an antioxidant or a prooxidant remains unclear.

To clarify the antioxidant ability of synephrine, we measured radical scavenging ability of synephrine against hydroxyl radical and superoxide anion radical using ESR. Our ESR results suggest that synephrine has the ability to scavenge both of hydroxyl radical and superoxide anion radicals (Fig. 3). We demonstrated for the first time the radical scavenging activity of synephrine using ESR.

Oxidative stress is caused by the disturbance in the balance (imbalance) between ROS production and antioxidant defenses [45, 46]. Oxidative stress is one of the key factors involved in several acute and chronic disease, such as cancer, alcoholic liver disease, diabetes, acute respiratory disease, Alzheimer’s disease, Parkinson’s disease and aging [47, 48]. Our ESR results suggest that synephrine may prevent oxidative stress-related diseases and senescence.

H_2_O_2_, which is produced by the one-electron reduction of superoxide, is not a free radical, but an active oxygen species. H_2_O_2_ can pass through the cell membranes, including the inner and outer membranes of the mitochondria, and react with iron (II), resulting in the production of hydroxyl radicals, which is called the Fenton reaction [29]. Hydroxyl radicals are highly ROS that oxidize biological components such as nucleic acids, proteins, and lipids [49, 50]. Zhong et al. described damage to macromolecules, including DNA, induced by hydroxyl and peroxyl radicals produced in H_2_O_2_-exposed cultured cells [51].

To verify the protective effect of synephrine against H_2_O_2_-induced premature senescence due to its antioxidant activity, we examined its effects on ROS production, lipid peroxidation, and oxidative DNA damage. In this study, we used an HPF fluorescent dye developed previously by our group. HPF specifically detects hydroxyl radicals and peroxynitrite at one-fourth the level of hydroxyl radicals [52, 53].

Significantly higher ROS generation was observed in the H_2_O_2_-treated cells using HPF, but synephrine treatment reversed this effect (Fig. 4). Furthermore, we showed that synephrine can scavenge hydroxyl radicals using ESR (Fig. 3). These findings suggest that synephrine suppresses the generation of hydroxyl radicals by the Fenton reaction. Our previous study showed a strong relationship among ROS, lipid peroxidation, and apoptosis after X-ray of a hepatocellular cell line [54]. HNE is a major marker of lipid peroxidation and originates from phospholipid-bound arachidonic acid. Monroe et al. suggested that the major end-products of lipid peroxidation, such as 4-hydroxynonenal (4-HNE), induce cellular senescence in IMR90 fibroblasts and murine adipose stem cells [55]. In this study, synephrine significantly reduced the lipid peroxidation induced by the H_2_O_2_ treatment, (Fig. 5). Moreover, oxidative damage occurs frequently when ROS attacks the free nucleotides of substrates for DNA synthesis and nucleotides within the DNA strand due to the oxidation of nucleotides that consist of DNA. 8-OHdG is one of the main types of oxidative DNA damages and is widely used as a marker. As shown in Fig. 6, oxidative DNA damage increased by H_2_O_2_ treatment. However, synephrine treatment significantly reduced the oxidative DNA damage in a concentration-dependent manner. Oxidative DNA damage activates various signal transductions pathways, resulting in cell cycle arrest, DNA repair, and apoptosis induction. These findings indicate that synephrine suppresses the cell cycle arrest caused by oxidative DNA damage. H_2_O_2_, including the inner and outer mitochondrial membranes, passes through the cell membranes. H_2_O_2_ enhances ROS generation, lipid peroxidation, and oxidative DNA damage, causing mitochondrial dysfunction.

Ziegler et al. reported that excessive mitochondrial ROS, impaired mitochondrial dynamics, electron transport chain defects, bioenergetic imbalance/increased AMPK activity, decreased mitochondrial NAD^+^/altered metabolism, and mitochondrial Ca^2+^ accumulation are important for cellular senescence [56]. These findings indicate that mitochondrial dysfunction is related to H_2_O_2_-induced premature senescence.

Mitochondria are the primary source of ATP, and mitochondrial dysfunction leads to reduced ATP production [57]. A decrease in mitochondrial membrane potential is also related to mitochondrial dysfunction, which leads to reduced ATP production [58]. Mitophagy refers to autophagy in the mitochondria and is a defense mechanism that prevents mitochondrial dysfunction-related diseases by selectively removing mitochondria damaged by oxidative stress or DNA damage [59].

We examined the effects of synephrine on mitochondrial membrane potential, ATP production, and mitophagy during H_2_O_2_-induced premature senescence and found a significant decrease in mitochondrial membrane potential (Fig. 7) and ATP production (Fig. 8) in H_2_O_2_-treated cells. In addition, an increase in mitophagy, which removes damaged mitochondria, was observed in Fig. 9. However, the significant effects of synephrine against the mitochondrial membrane potential (Fig. 7), ATP production (Fig. 8), and mitophagy (Fig. 9) were observed only in the 50 µM synephrine-treated cells. Although the effects of synephrine on mitochondrial function were not sufficiently observed in the 10 µM and 25 µM synephrine-treated cells, our results do indicate that synephrine restores mitochondrial function, thereby suppressing H_2_O_2_-induced premature senescence. However, other factors such as NAD^+^ and AMPK activity must be investigated to determine the relationship between mitochondrial dysfunction and H_2_O_2_-induced premature senescence. Furthermore, mitochondrial dysfunction promoted SASP secretion [60]. Further investigation of the correlation between mitochondrial function and SASP is required.

To clarify the mechanism of cell cycle arrest during cellular senescence, we measured the expression levels of key proteins (p53, p21, and p16^-INK4A^) involved in cell cycle arrest via western blotting. Activation of the p53–p21 and p16^-INK4A^–pRB pathways causes cell cycle arrest [61, 62]. The expression levels of p16^-INK4A^ and p21, which are cyclin-dependent kinases, are increased in response to DNA damage. Constitutive activation of the RB protein by high levels of cyclin-dependent kinases inhibits the transcriptional activity of E2F, a transcription factor essential for cell cycle progression from the G1 to S phase, thus resulting in cell cycle arrest [63].

Increased oxidative stress induces DNA damage, including base oxidization, single- and double-strand breaks, and telomere shortening, activating the p53 and pRB pathways, thereby causing cell cycle arrest and senescence [64, 65]. p53, p21, and p16^-INK4A^ expression levels were significantly increased in H_2_O_2_-treated cells but significantly reduced by synephrine treatment (Fig. 10). Accumulated mitochondrial ROS contributes to cellular senescence via the p53–p21 or p16^-INK4A^–pRB pathways [59, 63]. Therefore, mitochondrial dysfunction is possibly involved H_2_O_2_-induced premature senescence. This study suggests that synephrine inhibits oxidative stress and mitochondrial dysfunction, and also suppresses cellular senescence through the p53–p21 and p16^-INK4A^ pathways. However, the detailed mechanisms underlying these effects in vitro still remain unclear and further investigation is necessary for a more thorough understanding. For future development, it is necessary to conduct animal experiments to investigate the effects of synephrine in vivo and to deepen our understanding. Furthermore, clinical applications are expected in areas such as supplements aimed at preventing aging, sunscreens for UV protection, and protective gear to reduce oxidative stress caused by radiation.

## Conclusion

This study is the first to reveal the radical scavenging activity of synephrine. Furthermore, this study showed that synephrine inhibited H_2_O_2_-induced oxidative stress and mitochondrial dysfunction and suppressed SIPS by inhibiting the p53–p21 and p16^-INK4A^–pRB pathways. Overall, our results suggest synephrine as an effective therapeutic agent against cellular senescence and various oxidative stress-related diseases.

## Data Availability

No datasets were generated or analysed during the current study.
